# Male Fern in Chronic Tuberculous Affections

**Published:** 1908-12-26

**Authors:** 


### MALE FERN IN CHRONIC TUBERCULOUS AFFECTIONS.

Male fern is chiefly known for its anthelmintic properties. It is only recently that it has been found to be a valuable remedy in the treatment of torpid tuberculous affections of bones, joints, or lymphatic glands, and of that class of patients formerly styled scrofulous. It also seems to be of distinct service in certain cases of pulmonary phthisis, especially in young persons in whom there is no pyrexia.

The difficulty is, of course, to determine how much of the cure is produced by the drug given, and how much is due to the *vis medicatrix naturæ.* That the drug is itself responsible for some of the benefit that follows its administration is suggested by the fact that not every preparation of it is equally good: the oleo-resin seems to be the least satisfactory, whilst the ether-alcohol extract and a white wine maceration are both good. The latter is prepared by pulping the fresh root and steeping it in white wine; although very active, it is obvious that it cannot always be obtained when required. The extract, on the other hand, is always obtainable; it is prepared by percolating the dried drug first with ether and then with alcohol, and mixing the two residual extracts.

The best results appear to be obtained by administering the ether-alcohol extract in the form of pills containing about a grain and a half for adults and proportionately less for younger persons—say half a grain at 10 years of age, or a grain at 15 or 16. A medium dose would be two such pills per diem, and it is a good plan to give them for a fortnight at a time, with intervals of 10 days or so between each such period of administration. It is reported that, far from deranging digestion, appetite increases, the bowels are regular, and no untoward symptoms are to be anticipated; the chief points are to be sure that the drug has been collected at the time of its greatest activity, and that it has been dried at a low temperature without delay. Administration of the ether-alcohol extract in pill form obviates the difficulty of taste.

